# Conference report: current advances in embryo model research at the International Dutch Embryo Model Meeting 2025

**DOI:** 10.1242/bio.062189

**Published:** 2026-01-22

**Authors:** Jeske Strik, Joelle de Visser, Charis Fountas, Fieke Verhaaf, Danique Bax, Romy Geuvers

**Affiliations:** ^1^ Department of Molecular Biology, Faculty of Science, Radboud Institute for Molecular Life Sciences (RIMLS), Radboud University, Geert Grooteplein Zuid 26/28, 6525 GA Nijmegen, the Netherlands; ^2^ Developmental, Stem Cell and Cancer Biology, Swammerdam Institute for Life Sciences (SILS), University of Amsterdam, Science Park 904, 1098 XH Amsterdam, the Netherlands; ^3^ Division of Gene Regulation, the Netherlands Cancer Institute, Plesmanlaan 121, 1066 CX Amsterdam, the Netherlands; ^4^ Oncode Institute, 3521 AL Utrecht, The Netherlands; ^5^ Hubrecht Institute, Royal Netherlands Academy of Arts and Sciences (KNAW) & University Medical Center Utrecht, Uppsalalaan 8, 3584 CT Utrecht, the Netherlands

**Keywords:** Embryo model, Gastruloid, Blastoid, Pluripotency, Gametes, Bioethics

## Abstract

The International Dutch Embryo Model Meeting took place at Radboud University in Nijmegen, the Netherlands, on March 27 and 28, 2025. This year's meeting, which was previously held twice as a Dutch event, served as a platform to discuss the progress made in studying embryonic development using *in vitro* embryo-like structures. Organised into sessions on pluripotency, blastoids, gastruloids, and gametes, the meeting featured presentations by invited (inter)national speakers. These talks were complemented by shorter presentations from academics and industry professionals, as well as poster presentations interspersed between sessions. The meeting included an interactive ethics session that explored the opportunities and risks of researching embryos and embryo-like structures. This Meeting Review aims to provide an overview of the first International Dutch Embryo Model Meeting by highlighting the topics discussed by leading experts in embryo modelling.

## Teaser

**Summary:** The 2025 International Dutch Embryo Model Meeting was a high-quality conference, providing a broad perspective on embryo models and highlighting exciting new avenues for the future of the field.

## Introduction

The process from germ cell development through fertilisation to the formation of a complete embryo with the potential to develop into a healthy adult is a complex biological phenomenon. Although decades of extensive research using embryos from various model organisms have led to a better understanding of embryonic development, major knowledge gaps remain, particularly on developmental processes during the peri- and post-implantation stages in mammals.

These periods mark the onset of key developmental events such as the exit of pluripotency, gastrulation, and the start of organogenesis. However, the study of these processes is complicated due to the inaccessibility of the embryo. Furthermore, studying human embryos directly is limited by both practical and ethical constraints. Culturing human embryos *ex utero* beyond 14 days is legally prohibited in many countries. In addition, the availability of human embryos is limited, and their use for research, including potential manipulations, is ethically controversial. These limitations hinder the investigation of crucial stages in early development, highlighting the need for models that represent these critical biological processes.

A key advantage of using model systems is that they eliminate the need for natural embryos or animal models and allow the investigation of developmental events beyond the 14-day limit. Another major benefit is the opportunity for manipulation: mechanical, cellular, or molecular processes can be perturbed to gain insights that are otherwise ethically or technically unfeasible using natural embryos. A rapidly growing field within developmental biology is now focusing on the optimisation and application of embryo models. Several types of *in vitro* embryo models have been developed, including blastoids, gastruloids, and gamete models. These various models differ in developmental stage and tissue composition and recapitulate only a specific window of embryonic development. In addition, studies on the maintenance or loss of pluripotency in embryonic stem cells and induced pluripotent stem cells (iPSCs) are essential to further advance this field. This makes the application of embryo models for research on early embryonic processes, such as maintenance or exit of pluripotency and differentiation, increasingly relevant.

**Fig. 1.**
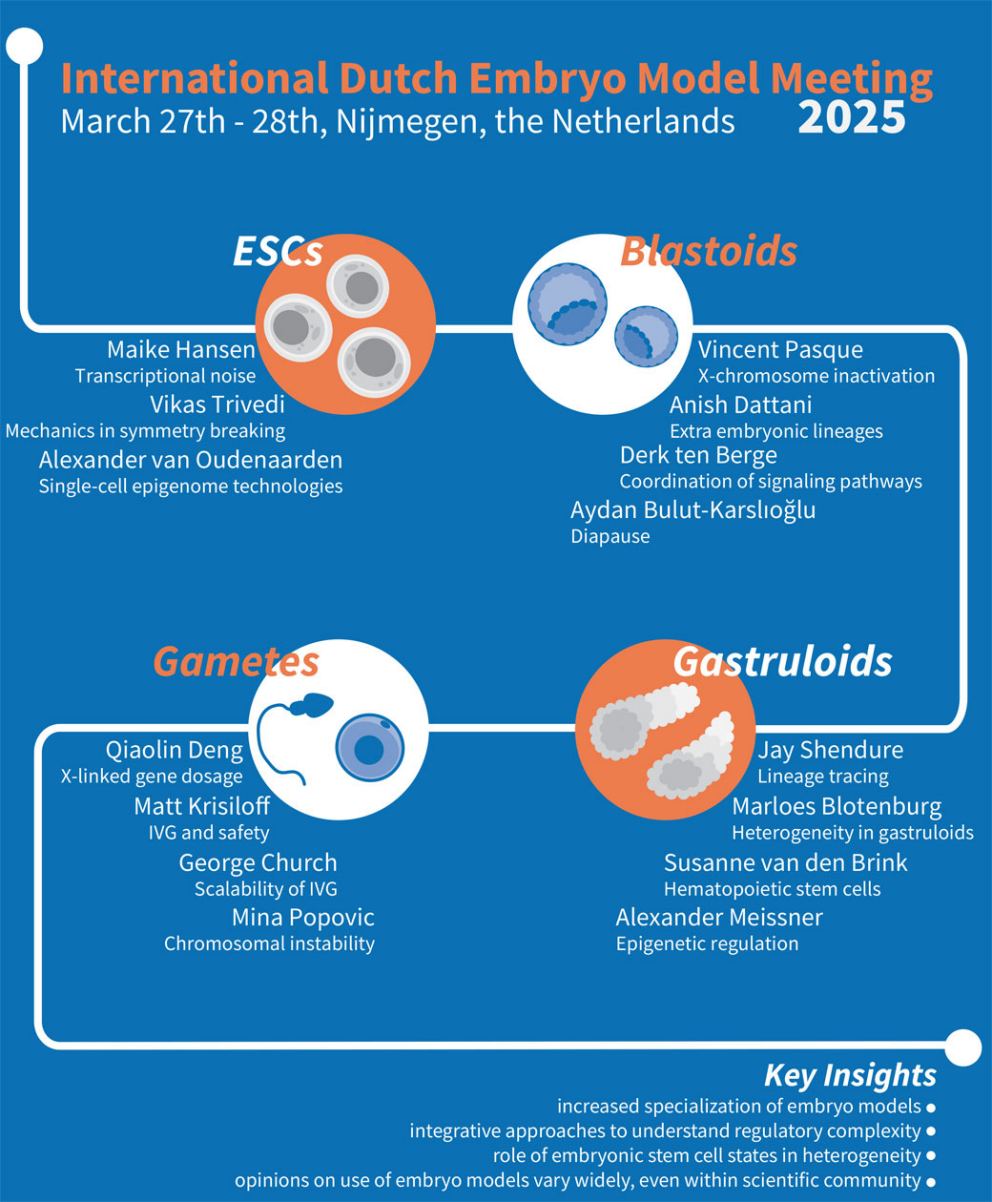
**Overview of the International Dutch Embryo Model Meeting 2025.** Schematic overview of thematic sessions at the International Dutch Embryo Model meeting and key insights.

The International Dutch Embryo Model Meeting is fully dedicated to research on these topics (Fig. 1). The meeting has previously been held twice as a successful Dutch national event, and due to increasing interest and enthusiasm, it has now been expanded into an international, multi-day conference. This year, the meeting took place in Nijmegen, hosted by the Radboud University (RU). The organisation was chaired by Dr Hendrik Marks, associate professor at the department of Molecular Biology and consisted of researchers from several institutes across the Netherlands: Dr Susana Chuva de Sousa Lopes [Leiden University Medical Center (LUMC), the Netherlands]; Dr Moritz Bauer (Hubrecht Institute, Utrecht, the Netherlands); Dr Luca Braccioli (Utrecht University, the Netherlands); Dr Niels Geijsen (LUMC, the Netherlands); Dr Ana Pereira Daoud (LUMC, the Netherlands) and Dr Derk ten Berge (Erasmus Medical Centre, Rotterdam, the Netherlands). Over the course of 2 days, invited, established scientists presented their research and early-career scientists were selected to provide a seminar. During lunch, there was ample time to visit the posters, which ranged from research about the oscillatory patterns of NOTCH signalling to computational modelling of blastoids. Furthermore, several companies showcased their latest products relevant to this field: BD, 10× Genomics, Vizgen, Miltenyi Biotec, Singleron, Promega, Bio-connect, Bio-Techne and Stemcell Technologies. The conference was sponsored by The Company of Biologists and ZonMw PSIDER project, a Dutch research program for which a large part is dedicated to advancing embryo models.

**Fig. 2.**
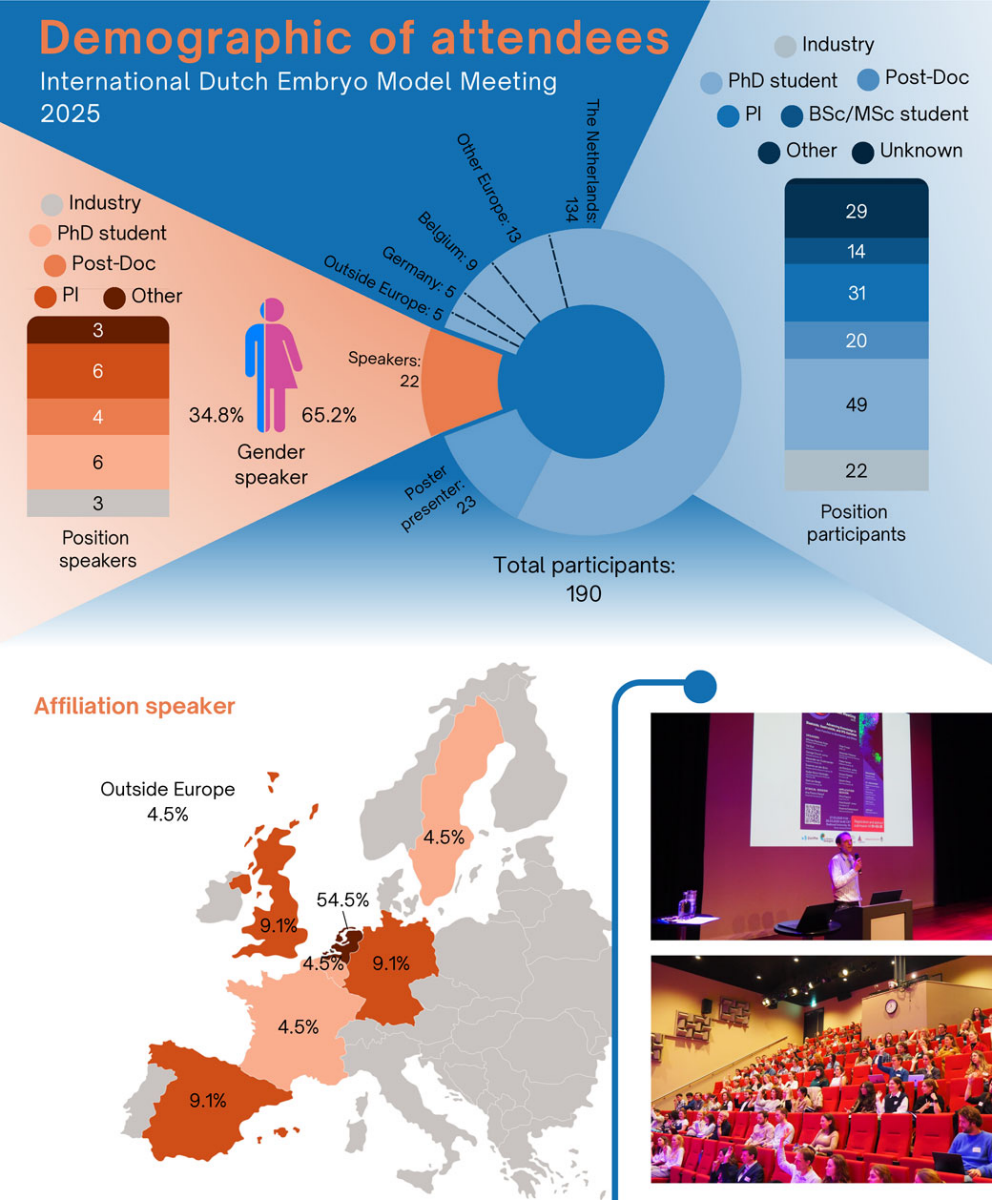
**Participant and speaker overview.** Distributions by career stage, gender and geographical location of affiliated institute, along with two representative photographs reflecting the conference setting. Photos were taken by Machiel Weistra.

To cover the major current topics of embryo model research, the keynote speakers and the selected short talks were organised into five thematic sessions. The first session focused on pluripotency, which is the foundation of development, as pluripotent cells are capable of forming all embryonic lineages. The second session was dedicated to blastoids, which model blastocyst development and implantation. This was followed by a session on gastruloids, modelling the gastrulation process until the initiation of organogenesis. The next session featured research on gamete models, which recapitulate gametogenesis. During the last session, ethical aspects of embryo models were discussed in an interactive debate. Together, these sessions provided a comprehensive overview of current advances and challenges in studying *in vitro* embryonic development and highlighted several state-of-the-art conceptual and technical frameworks in the field. This Meeting Review summarises the current topics and key insights gained during this meeting by showcasing a selection of the presented work and concludes with an outlook on emerging trends and future directions within the field of embryo model research.

## Summary of thematic sessions

### Pluripotency

Pluripotent cells are an essential part of embryology, as they have the ability to differentiate into the three embryonic lineages: endoderm, mesoderm and ectoderm. However, the regulation of these cell fate decisions and of the morphogenetic events needed to generate the different embryonic tissues remains only partially understood. To open the session, Prof. Dr Alexander van Oudenaarden (Hubrecht Institute, Utrecht, the Netherlands) introduced novel -omics tools to uncover these regulatory mechanisms. To capture the full complexity and heterogeneity of a cell population, it is essential to have single-cell resolution. While such techniques already exist to profile DNA accessibility and RNA expression, single-cell methods to investigate chromatin states are still relatively underrepresented. Four novel single-cell techniques were presented to study the epigenome, including sortChIC (Zeller et al., 2023) and TChIC (Zeller et al., 2024 preprint). Both techniques use cleavage-based genome mapping of chromatin-bound proteins, whereas TChIC also includes transcriptomic data. These tools can be used to better understand the chromatin landscape of pluripotent stem cells and to map the dynamics during embryonic development. In the following talk, Dr Maike Hansen (RU, Nijmegen, the Netherlands) explored the role of transcriptional noise in embryonic stem cells. Noise, defined as cell-to-cell variability in RNA or protein levels, is a well-documented event in these cells and has implications on developmental patterning, diversification and cell differentiation. Dr Hansen reasoned that there must be endogenous factors regulating noise if it can influence cell behaviour. To identify such regulators, her team conducted a comprehensive integrative screen combining single-cell RNA sequencing, proteomics, and regulatory enrichment analysis (García-Blay et al., 2025). This led to the identification of SON as a transcriptional noise modulator, and its knockdown lowered state-switching rates, suggesting that noise allows cells to explore different states. Concluding the session, Dr Vikas Trivedi (EMBL, Barcelona, Spain) presented his research on how embryonic stem cells can break symmetry during *in vitro* development. His lab focuses on the integration and the underlying crosstalk of multiple approaches, such as (epi-)genetics, mechanics and metabolism. Using the differentiation of pluripotent stem cells to form gastruloids (covered in more detail below), their research focuses on the formation and polarisation of the mesodermal T+ cells. He proposed a model in which T+ cells are formed by instructive signalling, potentially regulated by the metabolic state of the cells (Stapornwongkul et al., 2025). Once formed, differences in their mechanical properties guide the spatial organisation of T+ and T- cells (Oriola et al., 2024 preprint). Together, these talks emphasised the importance of using integrated approaches to uncover the different modes of regulation behind pluripotent stem cell function and differentiation.

### Blastoids

Blastoids model the early embryonic stage of the blastocyst, which in humans occurs about 5 to 7 days after fertilisation (Heidari Khoei et al., 2023). Both the blastocyst and the blastoid are composed of hypoblast or primitive endoderm, epiblast and trophectoderm cells. The blastoid is an excellent model for studying early cell fate decisions, X-chromosome inactivation, and implantation, occurring during the timeframe where many pregnancy failures are observed. Dr Vincent Pasque (KU Leuven, Belgium) opened the session and discussed several ongoing projects in his lab. The first part focused on the similarities and differences in key developmental processes between mouse and human. In human embryos, the extra-embryonic mesoderm arises before gastrulation, whereas in mice, it develops after the onset of gastrulation. However, in mice, X-chromosome inactivation is earlier (pre-implantation stage) than in humans (post-implantation). Furthermore, Dr Pasque demonstrated work on how naïve cells transition to primed cells, involving a key role for DNMT3L, without which blastoids cannot properly form all lineages. Along similar lines is the work of Dr Anish Dattani (Living Systems Institute, University of Exeter, UK), who discussed the tri-lineage potential of human naïve cells and how signalling pathways like MEK/ERK and FGF can unlock these lineages. Next to blastoids, other embryo models or combinatorial models were also discussed during the session. Inspired by the discrepancy in developmental robustness between embryos and embryo models, considering differentiation and morphogenesis, Dr Derk ten Berge (Erasmus Medical Centre, Rotterdam, the Netherlands) presented a morphogenetic model to study, among others, lumenogenesis and polarisation. One of the selected short talks (Asli Ak, Maastricht University, Maastricht, the Netherlands) demonstrated work on combining endometrial organoids and blastoids to study the intricacies of maternal-embryonal crosstalk. Finally, an intriguing aspect of embryogenesis that is often overlooked was presented by Dr Aydan-Bulut Karslioglu (Max Planck Institute, Berlin, Germany) with her work on diapause. Her lab has identified a connection between metabolism and dormancy and has demonstrated that it is possible to induce diapause in human blastoids, demonstrating a conserved feature of embryonic development. The blastoid session highlighted the many research possibilities provided by this and comparable models. Through tremendous community effort, labs are improving the reproducibility and robustness of this model even further (Choi and Moon, 2024). This allows their expanded use to gain novel insights into the blastocyst stage of embryonic development.

### Gastruloids

The third session of the meeting focused on gastruloids – three-dimensional assemblies derived from pluripotent stem cells that recapitulate gastrulation and germ layer formation. Like other embryo-like models, gastruloids offer a scalable and tractable platform for studying embryonic development and modelling disease. Dr Marloes Blotenburg (University of Lausanne, Switzerland) gave a talk focusing on the role of stem cell maintenance conditions in cell differentiation potential, and subsequent mouse gastruloid development heterogeneity (Blotenburg et al., 2025). Specifically, her findings suggest that different stem cell culture conditions affect the morphology, elongation and timing of gastruloids. The second part of the talk shifted the focus to the epigenetic changes that occur during gastruloid formation. T-ChIC enabled the readout of transcripts and histone modifications in the same cell in gastruloids, showing that different cell lineages display distinct epigenetic regulation and transcriptional states (Zeller et al., 2024 preprint). Prof. Dr Jay Shendure (University of Washington, USA) gave a talk focusing on the source of intra-batch gastruloid heterogeneity. To better understand the origin of this heterogeneity, his group generated monoclonal mouse gastruloids and performed lineage tracing using a novel DNA typewriter approach (Choi et al., 2022). Their results indicate that intercellular heterogeneity can be found as early as after the first cell division and that this informs later cellular states. This sheds light on how early heterogeneity already biases lineage specification. Next, Dr Susanne van den Brink (Hospital del Mar Research Institute, Barcelona, Spain) discussed her work on using gastruloids to generate hematopoietic stem cells. Preliminary (Ragusa et al., 2025) and unpublished data suggest that modifications to the standard gastruloid protocol can produce lineages capable of generating hematopoietic stem cells. This paves the way for gastruloids to be used in clinical applications as a new tool for hematopoietic stem cell production, which currently is an open challenge. Finally, short talks further focused on the epigenetic regulation and intercellular communication occurring during gastruloid development.

### Gametes

*In vivo* gametes, upon fertilisation, have the full potential to develop into a healthy embryo, including all extra-embryonic tissues. Although gamete models do not directly recapitulate the embryo itself, research into *in vitro* gametogenesis (IVG) using human iPSCs is closely related to the embryo models discussed earlier, as iPSC-derived gametes may become source material for generating embryo models through artificial fertilisation. The field primarily focuses on germ cell specification, cellular integrity and safety, and gonadal development. Dr Qiaolin Deng (Karolinska Institute, Solna, Sweden) presented her research focusing on the impact of X-linked gene dosage in germ cell specification, using cell lines derived from patients with Klinefelter syndrome, who have extra copies of the X chromosome (He et al., 2025). The findings show that the USP9X, encoded by an X-linked gene that escapes inactivation, upregulates SOX2 expression, suggesting a role in regulating progenitor competence during germ-like cell specification. During the meeting, Matt Krisiloff, CEO of Conception (Berkeley, USA), discussed the current state of IVG and what the critical safety checkpoints should be for pre-clinical safety. He expressed optimism that a proof-of-concept protocol may be available in the next few years but underscored the importance of clear regulations on safety and quality control. Dr George Church, co-founder of eGenesis (Cambridge, MA, USA), agreed on the importance of pre-clinical safety and further emphasised the necessity of scalability and cost reduction for biomedical techniques like IVG for future patients. The focus of current studies is not exclusively on the gamete itself, but extends to the somatic cells of the gonads, which play a pivotal role in sex determination and differentiation and provide the environment and signals for *in vivo* gamete development. Next, during a selected short talk, Arina Puchkina (Erasmus Medical Centre, Rotterdam, the Netherlands) presented her preliminary work on an *in vitro* differentiation model for Sertoli cells, the sex determining cell type in the male gonads. Using the ‘DCM time machine’ mouse model, a recently developed technique that allows retrospective tracing of gene expression and enhancer activity (Boers et al., 2023), she studied mouse progenitors of Sertoli cells. Upon combining bulk data with single-cell RNA sequencing datasets, she identified multiple progenitor cell types. Building on these insights, the current goal is to optimise the *in vitro* differentiation model to better understand molecular processes underlying Sertoli cell development.

### Ethics

The International Dutch Embryo Model Meeting also included an ethics session. Considering the increased political debate centred around embryos and embryo-like structures, getting a discussion started between researchers about ethics and public engagement was an interesting addition to the conference. Dr Nienke de Graeff and Dr Ana Pereira Daoud from the Bioethics department at the Leiden University Medical Centre (the Netherlands) led the sessions. The session was composed of three rounds of a statement, followed by a debate between the experts, and then concluded with a discussion with the audience. The statements were about a variety of integrated embryo model-related dilemmas, like “research with early human embryos should be limited because their potential to become persons is intrinsically valuable”. From the debate between the ‘pro’ and ‘con’ speakers, played by the ethical experts, it became clear that both sides had strong arguments, relating to a fundamental question in bioethics of when life begins and should be protected. This was also reflected in the standpoints of the audience, for all three statements, the audience was split approximately 50:50. This shows that even within the field, there is no consensus on ethical aspects related to embryo research. Notably, there was a strong consensus across all three debates that we should include the public in discussions about the course of embryonic research. In support, one of the main arguments was that the taxpayer should at least agree with the research their money is spent on. If we include the public in an early stage, the chances are higher that our findings will actually be implemented in policy.

## Key insights and the future of the field

Over the past few years, *in vitro* embryo model research has evolved into a highly dynamic and interconnected field. Each embryo model focuses on a specific window of embryonic development, and combined, they expand our global understanding of early development. Several key themes are emerging in this rapidly evolving field, as highlighted throughout the meeting.

An example of integrating knowledge is the increasing specialisation of models such as somitoids, hematopoietic-centred gastruloids, monoclonal gastruloids, assembloids and hybrids of organoids with embryo models. The novel models use the origins of one embryo model yet combine knowledge from other fields to specialise. As the field continues to grow, it is likely that the number of specialised models will expand further.

Another prominent focus is on the epigenetic landscape of both DNA marks and histone modifications during pluripotency and lineage specification. Multiple presentations showcased emerging tools and strategies to study epigenetic regulation across various cell types. In parallel, another clear emerging trend is the integration of both biological systems and technical approaches. Multi-scale techniques are becoming essential to understand the full regulatory complexity of embryogenesis. The integration of epigenomic, transcriptomic, metabolomic, proteomic or mechanical data will be necessary to deepen our understanding of the full regulatory landscape.

Despite these advances, heterogeneity remains a major challenge in the field. Significant variation exists between different laboratories, cell lines and experiments. This heterogeneity is a major challenge in the field that limits reproducibility and data interpretation. Multiple speakers emphasised the critical role of embryonic stem cell states, affected by preculture conditions prior to differentiation, on the observed heterogeneity in embryo models. Addressing this heterogeneity is essential to improve reproducibility and ensure robust data interpretation.

Finally, the ethical considerations of embryo model research were a topic of discussion. The meeting provided an opportunity for scientists to engage in the ongoing debate. Opinions varied widely, even within the scientific community. For a field advancing as fast as this one, it is crucial that ethical guidelines are clearly defined and can be reconsidered or adjusted in light of new scientific insights. This should go hand in hand with open communication within the field and to the general public so that the scientific community can, together with policy makers and the general public, reach a consensus. The next International Dutch Embryo Model Meeting will take place on 7-8 May 2026 in Utrecht, NL. Registration is open for all.
